# Adaptation and Validation of the Survey of Pain Attitudes (SOPA-Brief Scale (30 Items)) in Greek

**DOI:** 10.3390/jcm14082551

**Published:** 2025-04-08

**Authors:** Ioannis Dalakakis, Nadia Malliou, Despoina Sarridou, Eleni Moka, Aikaterini Amaniti

**Affiliations:** 1 Department of Anesthesiology and Intensive Care, School of Medicine, Aristotle University of Thessaloniki, 54124 Thessaloniki, Greece; idalakakis@gmail.com (I.D.); dsarridou@auth.gr (D.S.); aamaniti@auth.gr (A.A.); 2 Department of Anesthesiology, Creta Interclinic Hospital, Hellenic Healthcare Group (HHG), 71304 Heraklion Crete, Greece; mokaeleni@hotmail.com

**Keywords:** chronic pain, attitudes towards pain, survey of pain attitudes-brief, perceptions of pain, beliefs about pain

## Abstract

**Background**: The attitudes and beliefs of patients with chronic pain significantly affect their response to treatment. The Survey of Pain Attitudes (SOPA) scale was developed to identify pain-related beliefs. The aim of the present study was to adapt and validate the short version (30 items) of the Survey of Pain Attitudes in 200 Greek patients living with chronic pain, mainly due to rheumatic and musculoskeletal diseases (RMDs). **Method**: In addition to the SOPA-Brief scale (30 items), the participants completed the Pain Beliefs, Perceptions and Attitudes Inventory (PBAPI) and also the Chronic Pain Coping Inventory (CPCI). **Results**: Data analysis revealed that the internal reliability coefficient of the scale in the Greek language was Cronbach’s a = 0.773 for the individual items, and for the subscales, it ranged from Cronbach’s a = 0.56 (for the SOPAMedication scale) to Cronbach’s a = 0.78 (for the SOPASolicitude scale). Similarly, the SOPA-Brief subscales in Greek showed positive correlations with subscales of both the PBAPI and the CPCI. Finally, an exploratory factor analysis was performed on the dataset and confirmed the structure of the original scale (Eigenvalues > 1), with 71.54% of variance explained. **Conclusions**: Overall, the psychometric properties of the short version of the Attitudes Towards Pain Scale (30 items) in Greek show acceptable internal reliability and validity for the scale to be used in daily clinical and research practice.

## 1. Introduction

In recent years, research has increasingly been focusing on the psychosocial factors that influence pain and, by extension, affect the outcomes of treatment. The aim is to generate more data on the parameters of pain that may influence decision making on chronic pain management so that treatment planning can become even more effective [1]. An individual’s beliefs and attitudes towards pain can influence the likelihood of them developing long-term pain or disability associated with it [2]. It has been found that patients who adopt passive coping strategies, such as “rest and medication”, use three times as many healthcare appointments and have twice the level of disability due to their pain compared to those who adopt active strategies (e.g., exercise) [3]. Changing patients’ attitudes towards pain can reduce their level of pain and its impact on their quality of life [4].

It is not uncommon that patients who have experienced pain for prolonged periods of time try to avoid any activity that could potentially exacerbate their pain. This is a coping strategy that can become maladaptive when used repeatedly, leading to an avoidant behavior based on fear [5,6]. The assessment and use of coping strategies are the cornerstones of pain management. A patient may use cognitive and behavioral resources to manage specific external and/or internal demands that are assessed as stressful or go beyond the individual’s resources [7]. Pain coping strategies employ cognitive processes that involve an internal appraisal of the pain and then the mobilization of the coping resources available for managing the pain. This is followed by responses to pain and its effects based on the initial appraisals. Responses have been classified as active (e.g., remaining in employment) and passive (e.g., continued rest), depending on whether the response involves performing a task versus avoiding activity as a means of dealing with pain. Current pain adaptation paradigms do not account for the numerous patients that do not show signs of the effects of chronic pain. Resilience is the integrative perspective illuminating the traits and mechanisms underlying the ability to recover and the sustainability of quality of life in individuals recovering from trauma and distress [8]. Maladaptive pain coping and beliefs may eliminate any positive benefits of adaptive coping responses [7].

A person’s attitudes and beliefs have a significant impact on their response to pain treatment [9]. Multidisciplinary approaches to pain typically focus on cognitive interventions aimed at changing unhelpful or dysfunctional cognitive attitudes and beliefs about pain, such as avoidance and fear of activity, catastrophizing and the belief that pain is necessarily the result of tissue damage [10]. Studies have shown that these approaches lead to increased functional performance, induce positive changes in pain experience (i.e., measures of sensation and ratings of pain as “unpleasant”), increase cognitive coping and appraisal (positive coping measures) and decrease behavioral expressions of pain [11]. Several scales exist to assess patients’ attitudes and beliefs about pain. The Survey of Pain Attitudes (SOPA) is one of them, and it was developed to identify pain-related beliefs. It has been proven to be useful in the management of chronic pain [12,13]. Previous results of SOPA use beliefs of associating disability with pain and both psychological and physical dysfunction. A strong belief that pain equals physical injury in association with greater physical dysfunction and that emotions affect pain which is associated with psychosocial dysfunction [14].

This scale, which assesses a range of attitudes towards pain, was originally developed by Jensen et al. (1987) [13,14,15] to assess patients’ attitudes towards five pillars of the chronic pain experience: pain control, pain-related disability, medical treatment of pain, caring for others and medication for pain. In its original version, it consisted of 57 items and had seven subscales. Its factorial structure was not distinct or confirmed, and its completion time was quite prolonged. For these reasons, Tait and Chibnall (1997) [16] proceeded to create the short version, with 30 items, which was translated and evaluated for its psychometric characteristics in Greek in the present study. The reliability of the 30-item version of the scale was satisfactory (with a Cronbach’s range of a = 0.7 to 0.83; however, a was 0.5 in some subscales). The correlations of the final subscales with the subscales of the original version were high, and in addition, the corresponding correlations with other psychometric scales were also high. The SOPA-Brief (30 items) [16] consists of 30 questions with the possibility of a single response on a 5-point Likert-type scale, from 0 to 4, where 0 means completely wrong and 4 means completely right. The questionnaire can be completed either by the participant, the researcher or a health professional, if necessary. There are some additional scales that assess pain-related beliefs, such as the Pain Beliefs and Perceptions Inventory [17], the Pain Catastrophizing scale [18] and also the Fear–Avoidance Beliefs Questionnaire (FABQ) [19]. In the present study, we tried to adapt and validate the psychometric characteristics of the SOPA-Brief (30 items) [16] in Greek patients with chronic pain, mainly due to rheumatic and musculoskeletal diseases (RMDs).

## 2. Materials and Methods

For the use of SOPA Brief in this study, including the process of translation, adaptation and validation in Greek, permission was obtained from Professor Tait. The translation process lasted one month. The translation from the original language into the Greek language was performed by an independent translator. A bilingual translator adapted the questionnaire into Greek, his native language, in order to better reflect the local expressions. The first translator was aware of the specific concepts the questionnaire intended to measure, so that the Greek version would more closely reflect the original tool. The second translation was produced by a “naïve translator” who did not know the aim of the questionnaire, so that subtle differences with the original questionnaire could be identified [15]. Discrepancies between the two translators were discussed with the researcher and an expert in order for them to be resolved. The initial translation was performed independently to ensure the accuracy of the translation. Similarly, the reverse translation was performed by two independent translators, in their native language, to avoid any form of bias. Finally, the expert and the researcher cross-checked the result before proceeding to the pilot phase of weighting. For the process of the cross-cultural adaption of questionnaires, there are numerous guidelines, and several best practices have been suggested, but no one “gold standard” has, as of yet, been clearly defined [20]. Therefore, in this process, we followed the guidelines of Tsang et al. [21].

The questionnaires were distributed to a total of 200 patients with a diagnosis of chronic pain and a primary diagnosis of an RMD (68.5%), without any restriction or exclusion criteria relative to a specific diagnosis in the validation process. These were patients in the outpatient clinic of the Chronic Pain Clinic of the AHEPA University General Hospital of Thessaloniki, during the period June–December 2018 for the pilot phase (n = 50). Then, during the period January–June 2019, data collection from the remaining participants (n = 150) followed. Finally, during the period December–May 2023, the first 30 patients were recruited to complete the questionnaires again, in order to conduct test reliability checks for repeated measures.

The sampling method was convenience sampling. The chronic pain diagnosis criterion had to be met, and the researcher had to have easy access to a high enough number of patients. Although this is a non-probability sampling method, it is the most applicable and widely used method in clinical research. In this method, researchers approach participants according to their availability and accessibility. Therefore, it is quick, cheap and convenient for its accessibility and proximity to the participants [22]. Even though non-probability sampling methods are practical and cost-effective, they do present some disadvantages and biases, like selection bias because participants are not randomly chosen. This could result in some groups not being present in the sample and hence in the researcher not being able to achieve the necessary representativeness. This could limit the ability to generalize outcomes. Still, in our target population, this method is preferred due to the specific criterion that needs to be present [23].

In Chronic Pain Units, patients are referred for chronic-pain-specific follow-up regardless of their baseline diagnosis, which makes them suitable participants in this validation study. Still, this Pain Unit mainly treats patients with non-malignant chronic pain, due to fibromyalgia, osteoarthritis, rheumatoid arthritis and several other, rarer chronic pain syndromes.

During recruitment, participants were informed about the purposes of this study verbally and written consent was obtained, according to which they were informed (a) on the principles of the protection of their personal data and (b) that their personal data would be safeguarded. The principal researcher also had to keep records until the end of this study. Then, the consent form informed participants of the ways in which they could withdraw their participation at any time and contact the researcher by phone or email. An adequate time frame was kept in order to avoid response from memory for test–retest reliability.

### Psychometric Properties and Statistical Analysis

Each scale and questionnaire was evaluated based on two properties: reliability and validity. Questionnaire reliability was assessed through internal consistency and test–retest reliability. Internal consistency assesses the extent to which the questions that make up a scale measure the same concept. It is calculated by means of Cronbach’s alpha, which assesses the degree of correlation between the questions in the questionnaire. Values greater than or equal to 0.7 are acceptable; however, in the use of new scales and questionnaires, values between 0.5 and 0.6 could also be acceptable. Indeed, there is much debate, especially for scales and questionnaires undergoing weighting, whether researchers derive more information about the internal reliability of the new instrument by looking at Cronbach’s for all individual items or only for subscales when they exist [24]. In this study, item internal consistency was calculated for each question. Test–retest reliability was calculated using Spearman’s rho correlation coefficient, which is able to adopt values between −1 and 1, where 1 indicates a perfect correlation and −1 indicates a perfect negative correlation. In this study, a correlation was interpreted as very strong (r > ±0.8), moderately strong (r ± 0.6–0.8), fair (r ± 0.3–0.6) and poor (r < ±0.3) [25].

Exploratory factor analysis (EFA) was performed on the data to examine the covariance of variables by groups in order to interpret the correlations between them. Most importantly, this was to check whether the tool adapted to the Greek language follows the original structure of Tait and Chibnall [16] with 7 factors. If not, some structural adaptation, better suited for the Greek population, would be needed. The Kaiser–Meyer–Olkin (KMO) test and Bartlett’s global test were used to test the suitability of the collected data in applying the factor analysis. KMO values > 0.5 indicate the correct choice of method for the analysis [26]. The eigenvalue criterion (eigenvalue of >1.0), which is commonly adopted for factor extraction, was used to select the number of factors. Finally, a shift in axes to principal component analysis was applied. The validity of conceptual construction was examined by correlating the questionnaire with related questionnaires, and emphasis was placed on subscales and dimensions that showed a statistically significant correlation at both *p* < 0.05 and *p* < 0.01.

## 3. Results

### 3.1. Demographic Characteristics of Participants

The mean age of the participants was 65 years, with sd = 14.8 years. Of the participants, 63% were female and 37% were male. A total of 44.5% of the participants had completed secondary education and 29% had completed university education, while 7% had completed compulsory education and 19.5% were technical education graduates. From the 50 patients who participated in the pilot phase, 30 were females (60%) and 20 were males (40%). In total, out of 200 participants, 37% (n = 74) were males and 63% (n = 126) were females. In both phases of the validation process, the pilot phase and the main phase, in addition to the SOPA-Brief, the Pain Beliefs Attitudes and Perceptions Inventory (PBAPI) and the Chronic Pain Coping Inventory were administered.

Asymmetry and SD item evaluation analysis of the questionnaire showed that skewness values for questionnaire items were to the left/negative side, albeit ranging close to values from −0.5 to +0.5, indicating a normal distribution. Extreme skewness was marked for seven items [2,3,5,7,12,13,25]. These were items involved in several SOPA subscales and were not removed in this stage of the analysis following Tait and Chibnall’s initial structural use of the questionnaire, as well as other researchers’ approach [27,28].

### 3.2. SOPA Brief Internal Reliability Index (30 Items)

The internal reliability of the SOPA Brief (30 items) was found to be Cronbach’s a = 0.67 for all questions. For the individual scales, the index ranged from 0.506 (for the SOPAMedication scale) to 0.883 (for the SOPAEmotion scale). [Note that the scale has reversed items.] In the second phase, when the remaining patients in this study (n = 150) were included, the internal reliability of the SOPA Brief was calculated for all items of the scale and was Cronbach’s a = 0.773 for the individual items (after reverse coding). For the subscales of the questionnaire, it ranged from 0.56 (for the SOPAMedication scale) to 0.78 (for the SOPASolicitude scale) (Table A1).

### 3.3. Reliability Index of Repeated Measures

In the third phase, the first 30 patients were given the SOPA-Brief and PBAPI questionnaires again after a long period of time to determine the test–retest reliability index. The original study design planned for a long interval of 3 months, suited for less stable constructs such as beliefs and attitudes [29]. The actual time interval was a lot longer than that. The back-to-back lockdowns following strict national health protocols in Greece due to COVID-19 pandemic resulted in time deviations from the original study design, and the test–retest sampling was performed a year later. The analysis was performed using Spearman’s rho correlation coefficient, with the values being interpreted as very strong (r > ±0.8), moderately strong (r ± 0.6–0.8), fair (r ± 0.3–0.6) and poor (r < ±0.3). The SOPATreatment subscale had a r = 0.813, *p* < 0.01, the SOPAControl r = 0.996, *p* < 0.01, the SOPAHarm r = 0.963, *p* < 0.01, the SOPADisability subscale r = 0.484, *p* < 0.01, the SOPAMedication r = 534, *p* < 0.01, the SOPASolicitude r = 465, *p* < 0.01 and the SOPAEmotion r = 433, *p* < 0.01. Three of the subscales showed very strong reliability, whereas the other four subscales presented fair test–retest reliability.

### 3.4. Correlations with the Pain Beliefs and Attitudes Inventory and the Chronic Pain Coping Inventory

The SOPA Brief demonstrated positive correlations with some subscales of the Pain Beliefs and Attitudes Inventory-PBAPI and some subscales of the Chronic Pain Coping Inventory-CPCI, at both levels of statistical significance *p* < 0.05 and *p* < 0.01. In particular, the Solicitude subscale was found to be weakly positively correlated with the Mystery subscale and moderately positively correlated with the Self-Blame subscale of the PBAPI (Pearson’s r = 0.256 *p* < 0.01 and r = 0.351 *p* < 0.01, respectively). The Emotion subscale had a weak positive correlation with the Time and Mystery subscales of the PBAPI (r = 0.165 *p* < 0.05 and r = 0.247 *p* < 0.01). This subscale also had a weak positive correlation with the Social Support subscale of the CPCI (r = 0.146 *p* < 0.05). Furthermore, the SOPA Brief Control subscale had a weak negative correlation with the Time subscale (r = −0.252 *p* < 0.01) and Mystery subscale (r = 0.153 *p* < 0.05). The Medication subscale presented a weak positive correlation with the Self-Blame subscale of the PBAPI (r = 0.151 *p* < 0.05) (see Table A2 and Table A3).

### 3.5. Exploratory Factor Analysis (EFA)

The entire sample of data was checked for suitability to conduct exploratory factor analysis. To proceed with the analysis, it is necessary to meet the criterion of KMO > 0.5. For this dataset, the following was the case: the Kaiser–Meyer–Olkin Index (KMO) was calculated to be 0.632 (>0.5), which was considered sufficient for performing factor analysis on this dataset. Similarly, Bartlett’s test of sphericity was statistically significant (*p* < 0.01), indicating that the choice of factor analysis was correct.

The exploratory factor analysis (EFA) was performed to reduce the number of items, as this questionnaire has already been adapted and validated in other languages and in some cases, it was necessary to adapt (and assimilate) a subscale for optimal adaptation to the target population. The application of the factor selection criterion (eigenvalue > 1) resulted in seven factors, exactly as in the original version of the questionnaire [16]. Table A4 shows the factors with eigenvalues above 1 (eigenvalue > 1), as well as the percentage of covariance they explain. Note that the resulting factors (subscales) explain 71.54% of the covariance of the individual items. Finally, Table A5 shows the resulting loadings per factor.

## 4. Discussion

The results of this study showed that the adaptation and validation of the SOPA-Brief presents with similar psychometric characteristics as the original version of Tait and Chibnall [16], consistent with its subscales and reliability and validity measures, respectively. The internal reliability of individual items and the seven subscales were satisfactory, with two less-than-satisfactory subscales, like the original version of the questionnaire. Furthermore, the SOPA-Brief had strong, positive correlations with several subscales with similar conceptual constructs from other scales, such as the PBAPI and CPI. Most importantly, the EFA came up with a similar structure for this set of data, validating that the subscales work well with the Greek target population as well. The two weak subscales, SOPAMedication and SOPADisability, had already been highlighted in the original work of Tait and Chibnall [16], and the possible explanation for their non-satisfactory internal reliability might be their number of items or their conceptual overlap with other subscales. This needs to be further investigated in a future multicenter study in Pain Unit patients, probably resulting in a modified version of the SOPA-Brief. In a corresponding study on the adaptation and weighting of the SOPA-B in the Portuguese language, the two scales were removed as they were not supported by the factor structure of the analysis performed [30]. In the present case, the removal of the two subscales did not improve the model. The internal consistency of the model was already satisfactory.

There are several advantages associated with the availability of a short scale measuring pain-related attitudes specifically in clinical context. Clearly, the primary one relates to the ease of administration but also to the usefulness of the information extracted from the interpretation of the scores on the subscales indicating patients’ attitudes towards pain. Other advantages may arise from making it easier to provide a short scale assessing patients’ attitudes, which can be tested in a wide range of treatment settings (e.g., work empowerment programs), to identify patients whose attitudes might interfere with a good response to the treatment in question.

The present study supports the conclusion that the Survey of Pain Attitudes (SOPA-Brief) is a reliable and valid tool in Greek and can be used in the Greek population. However, the limitations of this study should be mentioned. One limitation is that data were collected at a very difficult time for conducting studies, with some time intervals longer than the ones in the original design, due to the COVID-19 pandemic and all the restrictions it created. Until cross-validation research is conducted on the scale in Greek, it is too early to assume that it is as reliable and valid as it appears to be in its original form in English. Similarly, it could be potentially premature to assume that the SOPA Brief possesses the clinical value of the equivalent scale in English, the Survey of Pain Attitudes (SOPA, 57 items) [28]. Another drawback of the present study concerns the fixed order in which the questionnaires were given. The order was designed to start with the basic demographic data of the participants and finish with the scales and questionnaires to be used in the validation process. However, the participants (n = 30) who took part in the repeated test were not given one of the selected questionnaires for the sake of brevity. These effects may account for the improvements in the internal reliability values of the scale and merit further investigation in future cross-validation research of the psychometric properties of the scale. Finally, another limitation is that the SOPA Brief had weak positive, one weak negative and one moderate positive correlation to the PBAPI and CPI subscales, and this is another limitation indicating the possible inherent differences in the basic constructs of the questionnaires.

Despite the aforementioned limitations, the Survey of Pain Attitudes—30 items (SOPA-Brief) appears to be a feasible assessment tool for everyday clinical practice, either as a stand-alone test or as part of a battery of tools measuring and assessing the psychological dimensions of pain. A positive feature of the scale is its brevity and ease of use, and with further study, it may emerge as a useful tool for future research on patient attitudes and beliefs, flexible enough to be used by a variety of disciplines involved in chronic pain management.

## 5. Conclusions

In regards to the psychometric characteristics of the SOPA-Brief in the Greek language, the findings of this study show evidence of acceptable internal consistency for this tool to be used in Greek pain patients. As mentioned above, it is expected for tools that have not been widely used in clinical settings to result in a Cronbach’s a ranging from 0.5 to 0.6 and still be considered acceptable for use. In our case, that only occurred for two out the seven subscales, same as for the original version of the SOPA-Brief. In addition, the tool appears to be comparable in terms of validity to those already existing and used in the Greek clinical context. In summary, the use of the SOPA-Brief in the Greek language can contribute substantially to chronic pain research and clinical practice as well. Its use can provide another validated tool for chronic pain assessment in Pain Units, therefore making it more possible to address patients’ needs for pain diagnosis, treatment and management.

## Data Availability

Data are available from the corresponding author on reasonable request.

## Abbreviations

The following abbreviations are used in this manuscript:

SOPA	Survey of Pain Attitudes
CPCI	Chronic Pain Coping Inventory
PBAPI	Pain Beliefs, Perceptions and Attitudes Inventory

## Appendix A

**Table A1:** Internal relevance indicators of SOPA Brief subscales in Greek.

Survey of Pain Attitudes—Brief Version (30 Items)		
	Cronbach’s a	
	Validation Phase	Test–Retest
Scale	n = 200	n = 30
Solicitude	0.78(5)	0.97(5)
Emotion	0.71(4)	0.99(4)
Control	0.69(5)	0.86(5)
Treatment	0.63(5)	0.87(5)
Medication	0.56(3)	0.64(3)
Harm	0.65(5)	0.98(5)
Disability	0.63(3)	0.79(3)

**Table A2:** Correlations of SOPA Brief subscales in Greek with CPCI subscales.

	Correlations SOPA-Brief with CPCI		
	SOPA-Brief Subscales		
CPCI Subscale	Emotion	Treatment	Disability
CPCIAssistance		0.154 *	
CPCISocialSupport	0.146 *		0.154 *
CPCICopingSelfStatements			0.163 *

* *p* < 0.05; ** *p* < 0.01.

**Table A3:** Correlations of SOPA Brief subscales in Greek with PBAPI subscales.

	Correlations SOPA-Brief with PBAPI					
	SOPA-Brief Subscales					
PBAPI Subscale	Control	Harm	Solicitude	Emotion	Medication	Disability
PBAPITime	−0.252	0.197 **				0.286 **
PBAPIMystery	0.153 *		0.253 *	0.165 *		
PBAPISelf-Blame			0.351 *	0.247 **	0.151 *	0.142 **

* *p* < 0.05; ** *p* < 0.01.

**Table A4:** Exploratory factor analysis (extraction method: principal component analysis).

Total Variance Explained									
Component	Initial Eigenvalues			Extraction Sums of Squared Loadings			Rotation Sums of Squared Loadings		
	Total	% of Variance	Cumulative %	Total	% of Variance	Cumulative %	Total	% of Variance	Cumulative %
1	5.649	18.830	18.830	5.649	18.830	18.830	5.136	17.119	17.119
2	5.271	17.569	36.399	5.271	17.569	36.399	4.008	13.360	30.478
3	3.251	10.838	47.237	3.251	10.838	47.237	3.641	12.138	42.616
4	2.723	9.075	56.313	2.723	9.075	56.313	3.215	10.717	53.333
5	2.275	7.582	63.894	2.275	7.582	63.894	1.938	6.459	59.791
6	1.255	4.182	68.076	1.255	4.182	68.076	1.888	6.294	66.086
7	1.040	3.467	71.543	1.040	3.467	71.543	1.637	5.457	71.543

**Table A5:** Exploratory factor analysis (principal component analysis—rotated component matrix).

Rotated Component Matrix ^a^							
	Emotion	Control	Harm	Solicitude	Treatment	Disability	Medication
1. There are many times I can influence the amount of pain I feel	**0.373**	0.299	0.051	−0.544	−0.035	**0.332**	0.026
2. I will probably always have to take pain medication	0.121	−0.100	0.011	**0.778**	0.087	−0.026	0.129
3. When I hurt, I want my family to treat me better	0.113	0.014	−0.196	**0.831**	0.076	0.056	0.195
4. I don’t expect a medical cure for my pain	−0.106	−0.022	**0.375**	0.211	**0.318**	−0.363	**0.501**
5. I have had the most relief from the pain with the use of medications	−0.075	**0.376**	**0.317**	**0.383**	−0.196	0.215	**0.469**
6. Anxiety increases the pain I feel	**0.493**	−0.202	**0.517**	0.277	0.145	0.194	0.044
7. When I am hurting, people should treat me with care and concern	0.250	−0.221	0.023	**0.763**	−0.254	0.107	−0.011
8. I have given up my search for the complete elimination of my pain through the work of the medical profession	0.168	0.253	−0.388	−0.217	**0.585**	0.017	0.021
9. It is the responsibility of my loved ones to help me when I feel pain	**0.809**	0.157	−0.190	0.259	0.007	−0.262	−0.032
10. Stress in my life increases my pain	**0.796**	−0.131	**0.310**	−0.152	−0.231	−0.007	−0.038
11. Exercise and movement are good for my pain problem	−0.064	0.127	**0.814**	−0.105	−0.072	0.227	−0.173
12. Just by concentrating or relaxing, I can ‘take the edge’ off my pain	0.156	**0.515**	0.278	−0.287	**0.306**	0.185	0.099
13. Medicine is one of the best treatments for chronic pain	−0.105	0.195	**0.358**	−0.014	0.132	**0.598**	−0.077
14. My family needs to learn how to take better care of me when I am in pain	**0.837**	0.132	−0.183	0.278	0.093	−0.096	0.001
15. Depression increases the pain I feel	**0.872**	0.035	0.125	0.047	0.135	0.219	−0.191
16. If I exercise. it can make my pain problem much worse	−0.117	−0.195	−0.322	0.125	0.202	−0.161	**0.653**
17. I believe that I can control how much pain I feel by changing my thoughts	0.268	**0.492**	−0.043	−0.200	0.212	0.157	−0.448
18. Often I need more tender loving care than I am now getting when I am in pain	**0.775**	−0.018	−0.168	**0.328**	0.096	0.095	−0.119
19. Something is wrong with my body that prevents movement and exercise	**0.695**	−0.049	−0.208	−0.080	0.123	−0.035	**0.319**
20. I have learned to control my pain	0.004	**0.838**	0.005	−0.057	−0.024	−0.071	−0.014
21. I trust that the medical profession can cure my pain	0.041	0.166	0.125	0.061	−0.255	0.769	−0.088
22. I know for sure that I can learn to control my pain	0.021	0.826	0.023	0.083	0.028	0.150	−0.123
23. My pain does not stop me from leading a physically active life	0.117	0.610	0.431	−0.216	0.044	0.129	−0.031
24. My physical pain will never be cured	0.192	−0.467	0.030	0.188	0.278	0.322	0.433
25. There is a strong relationship between my emotions and my level of pain	0.713	−0.131	0.246	−0.245	−0.225	−0.003	−0.120
26. I can do almost everything just as well as before my pain problem	−0.072	0.670	0.224	−0.282	0.192	0.050	−0.121
27. If I don’t exercise regularly. my pain problem will continue to get worse	−0.093	0.151	0.882	0.053	0.125	−0.016	−0.026
28. Exercise can reduce the amount of pain I experience	0.059	0.113	0.774	−0.207	−0.087	0.108	0.107
29. I am convinced that there is no medical procedure that will help my pain	−0.036	−0.029	0.152	0.097	0.861	−0.165	0.129
30. My pain would stop anyone from leading an active life	0.282	−0.666	0.037	−0.058	0.225	−0.369	−0.105
Extraction Method: Principal Component Analysis. Rotation Method: Varimax with Kaiser Normalization.a							
a. Rotation converged in 7 iterations.							
